# Temporal dynamics of alpha-band cortico-cortical coherence during motor preparation for successful and unsuccessful football penalty kicks

**DOI:** 10.3389/fpsyg.2026.1949955

**Published:** 2026-09-23

**Authors:** Guo Lu, Ming-Yang Cheng, Dan Li, Hatem Elbanna, Kuo-Pin Wang

**Affiliations:** 1 International Football Education School, Jilin Agricultural University, Changchun, Jilin, China; 2 Department of Sports Science Research, Taiwan Institute of Sports Science, Kaohsiung, Taiwan; 3 College of Leisure and Social Sports, Jilin Sport University, Changchun, China; 4 Department of Sports Psychology, Faculty of Sport Science, Mansoura University, Mansoura, Egypt; 5 Department of Sports Management, School of Business, ESLSCA University, Giza, Egypt; 6 Center for Cognitive Interaction Technology (CITEC), Bielefeld University, Bielefeld, Germany; 7 Master Program of Sport Facility and Health Promotion, National Taiwan University, Taipei, Taiwan; 8 NTU Plus Academy, National Taiwan University, Taipei, Taiwan

**Keywords:** attention, EEG coherence, motor performance, motor preparation, neural efficiency, psychomotor efficiency

## Abstract

**Introduction:**

Successful motor performance may depend on the efficient coordination of cognitive and motor processes during movement preparation. Although previous electroencephalographic studies have linked alpha-band (8–13 Hz) coherence with performance in upper-limb precision tasks, little is known about cortico-cortical communication preceding successful lower-limb actions.

**Methods:**

Sixteen skilled football players performed penalty kicks under an individualized task-difficulty condition. EEG coherence was calculated for eight electrode pairs connecting the left and right temporal regions with frontal, central, parietal, and occipital midline sites. Coherence was examined during an early preparation period from −2,000 to −1,000 ms and a late preparation period from −1,000 to 0 ms before kick initiation.

**Results:**

Successful kicks showed significantly lower Fz–T7 coherence than unsuccessful kicks across both preparation periods. Successful kicks also exhibited lower Cz–T8 coherence during the late preparation period.

**Discussion:**

Lower Fz–T7 coherence may reflect reduced interaction between verbal–analytical processing and motor planning, whereas lower Cz–T8 coherence may indicate reduced coupling between visuospatial processing and motor-control processes immediately before kick initiation. These findings extend previous evidence from upper-limb precision tasks to football penalty kicking and support the psychomotor efficiency hypothesis. Successful kicking appears to be characterized by selective and time-dependent reductions in functional coupling rather than a general decrease in cortical connectivity.

## 1 Introduction

In sport, the ability to adapt to task difficulty is essential for successful performance. As task demands increase, this adaptive ability requires athletes to allocate attention efficiently to task-relevant information while preventing excessive conscious control of movement (Cooke et al., 2014, 2015; Wang et al., 2019). This balance between efficient attention allocation and automatic yet adaptable movement control is particularly important in football penalty kicking. Successful kicking requires players to maintain goal-directed visual attention while resisting distraction from salient stimuli (Wilson et al., 2009), integrate visuospatial and proprioceptive information into coordinated lower-limb motor behavior (Giustino et al., 2024) before the action, and avoid excessive conscious control that may constrain movement automaticity (Niźnikowski et al., 2025). Importantly, task difficulty should be distinguished from psychological pressure. Task difficulty primarily increases the cognitive and motor demands required for successful execution, whereas pressure may redirect attention toward threat-related, evaluative, or self-referential information, thereby disrupting otherwise functional motor-preparation processes (Li et al., 2025; Slutter et al., 2021; Wilson et al., 2009). Although increased task difficulty may disrupt the automaticity of skilled performance (Wulf, 2013), successful performance appears to depend on the selective engagement of task-relevant cognitive and motor processes without excessive recruitment of processes unrelated to action execution (Babiloni et al., 2008; Cheng et al., 2017; Cooke et al., 2014; Li et al., 2025; Wang et al., 2019).

The psychomotor efficiency hypothesis provides a theoretical framework for understanding this adaptive ability, proposing that superior motor performance is characterized by the selective activation of essential neural processes and the inhibition of non-essential neural processes during motor preparation (Hatfield, 2018; Hatfield and Hillman, 2001). Consistent with this perspective, the MAP model further proposes that successful motor performance under challenging conditions is characterized by the efficient allocation of attentional resources to the core components of the action that directly support action execution (Bertollo et al., 2016; Bortoli et al., 2012). Supporting these theoretical accounts, previous electroencephalographic (EEG) studies have demonstrated that successful performance is associated with specific patterns of neural activation, as reflected in event-related desynchronization/synchronization and spectral power analyses. For example, successful performance in precision visuomotor tasks such as golf putting has been associated with greater alpha-band (range between 8 and 13 Hz) desynchronization or lower power over frontal and central regions before movement. These patterns have been interpreted as greater functional engagement of neural processes involved in motor planning and motor control (Babiloni et al., 2008; Cooke et al., 2014; Wang et al., 2019). Extending these findings to football, successful penalty kicks have recently been characterized by lower 8–13 Hz power over frontal and central regions during motor preparation, providing preliminary evidence that neural mechanisms previously identified in upper-limb-dominant precision tasks may also generalize to lower-limb-dominant sport actions (Li et al., 2025).

Although these findings highlight the importance of regional neural activation, EEG power analysis does not explain how cortical regions interact or communicate during motor preparation (Hatfield, 2018; Li et al., 2025). In addition to localized activation, successful performance depends on the functional coordination between cognitive and motor processes (Wang et al., 2021; Wang et al., 2022a). To capture this, electroencephalographic coherence provides a measure of cortico-cortical communication by quantifying the degree of covariation between activity recorded at two electrode sites (Hatfield, 2018; Wang et al., 2021). Within this framework, higher coherence indicates greater consistency of oscillatory activity, whereas lower coherence indicates less consistency of oscillatory activity, suggesting more independent activity between the recorded sites. Importantly, neither higher nor lower coherence is inherently advantageous; its functional meaning depends on the regions involved, the processing demands of the task (Deeny et al., 2003, 2009; Del Percio et al., 2011; di Fronso et al., 2018). Therefore, coherence analysis offers a complementary perspective by revealing how neural processes are coordinated, thereby providing additional insight into the neural mechanisms underlying successful motor performance beyond regional power analysis.

Building on this perspective of EEG coherence, previous studies have emphasized the importance of alpha-band communication between temporal and midline cortical regions during skilled motor performance, especially in shooting (Cheng et al., 2017, 2023; Deeny et al., 2003; Del Percio et al., 2011) and golf putting tasks (Babiloni et al., 2011; Wang et al., 2020; Wang et al., 2021). Within this network, the left temporal region (T7) has been associated with verbal-analytical processing, whereas the right temporal region (T8) has been associated with visuospatial processing (Deeny et al., 2003; Haufler et al., 2000). These temporal regions interact with midline frontal, central, parietal, and occipital regions, which are commonly linked to motor planning, motor control, somatosensory-parietal integration, and visual processing, respectively (Wang et al., 2022a). Empirical evidence from skilled marksmen, golfers, and dart throwers has shown that reduced alpha coherence between T7 and frontal or central regions has associated with superior motor performance, suggesting reduced verbal-analytical interference with motor planning and motor control processes (Cheng et al., 2017; Deeny et al., 2003, 2009; Lo et al., 2019). In contrast, coherence involving the right temporal region, particularly T8–parietal connections, may reflect visuospatial processing that supports goal-directed action during motor preparation (Del Percio et al., 2011).

Despite these advances in understanding neural coordination, the dynamic cortico-cortical communication underlying successful and unsuccessful football penalty kicking remains unclear. Existing EEG coherence research has primarily focused on upper-limb precision tasks (e.g., shooting, dart throwing, and golf putting; Babiloni et al., 2011; Cheng et al., 2017; Deeny et al., 2003, 2009; Lo et al., 2019; Wang et al., 2022a,b), meaning the findings may not fully generalize to lower-limb movements in sports such as football kicking (Del Percio et al., 2019; Li et al., 2025). Although recent evidence has observed regional frontal and central activation patterns preceding successful football penalty kicks, it remains unknown whether successful kicking is also characterized by specific patterns of cognitive-motor coupling. This gap is also consistent with previous calls to incorporate alpha-band functional connectivity into neurophysiological models of football-specific information processing (Del Percio et al., 2019).

To address this gap, the present study aims to examine alpha-band cortico-cortical communication during motor preparation for successful and unsuccessful football penalty kicks performed under an individual task difficulty condition designed to maintain a success rate of approximately 40%–60% (Li et al., 2025). To capture communication within the temporal–midline network, EEG coherence is calculated for eight electrode pairs linking left and right temporal regions with frontal, central, parietal, and occipital midline regions. The two preparation periods are distinguished to examine whether temporal–frontal and temporal–central communication contributed at different stages of action preparation. Based on previous evidence indicating reduced verbal-analytical, motor planning, and motor control interference during successful precision performance (Cheng et al., 2017, 2023; Deeny et al., 2003; Del Percio et al., 2011; Wang et al., 2022a), we hypothesized that successful penalty kicks would be characterized by lower 8–13 Hz coherence at T7–Fz during early motor preparation and lower 8–13 Hz coherence at T7–Cz during late motor preparation compared with unsuccessful kicks. The remaining electrode pairs were examined to explore whether visuospatial, parietal, and occipital communication also differentiated successful from unsuccessful penalty kicking.

## 2 Materials and methods

### 2.1 Participants

The required sample size was estimated using G*Power 3.1.9.7. The calculation assumed an alpha level of 0.05, statistical power of 0.80, and an effect size of *f(V)* = 1.25, corresponding to a partial *η*^2^ of 0.61 based on Li et al. (2025). The analysis included one group, 16 repeated measurements (2 performances × 8 coherence pairs), and a nonsphericity correction of 1. The Cohen in the options bar was chosen for representing output parameters. The resulting minimum required sample size was 13 participants. Given concerns regarding small-sample bias in EEG research (Albers and Lakens, 2018; Algermissen and Mehler, 2018), we recruited 16 male skilled football players (mean age = 24 ± 2 years, mean height = 179 ± 5.22 cm, mean training frequency = 3 ± 4 times per week). Participants were classified as skilled players because they had an average of 12.56 ± 2.50 years of playing experience (range = 8–17 years) and had regularly participated in regional-level competitions in Germany for at least 5 years. All of the recruited participants met the following criteria (Giustino et al., 2024; Li et al., 2025): (a) right-handed and right-footed; (b) normal or corrected-to-normal vision; (c) no history of neurological disease or use of medication affecting the central nervous system; (d) no injury at the time of enrolment in the study; and (e) no injuries in the 6 months prior to enrolment in the study. Participants also completed the Trail Making Test Part A (TMT-A) to screen for potential deficits in visual selective attention, which may influence visuomotor processing during the penalty-kick task (Lezak et al., 2004; Partington and Leiter, 1949). All participants provided written informed consent before participation. The study was approved by the Research Ethics Committee of Bielefeld University (No. 2021-044).

### 2.2 Study design

This study employed a within-subject study design to investigate the neuromotor processes underlying penalty kick performance in skilled football players. EEG data were recorded during motor preparation (2 seconds before action) to examine differences in neural activation between successful and unsuccessful penalty kicks under a difficult task condition.

### 2.3 Study measures

#### 2.3.1 Football penalty task

Participants completed a football penalty-kicking task based on the protocol outlined by Li et al. (2025), using a regulation Size 5 football (diameter = 23 cm; circumference = 70 cm). Each kick was taken from a distance of 1,100 cm toward a goal measuring 450 cm in width and 250 cm in height. A square target was placed within the goal area to determine successful attempts. All participants kicked with their dominant foot (right foot) and were allowed to use their preferred run-up length and approach angle. The warm-up session began with a 70 × 70 cm target in the goal, followed by five initial kicks. If the success rate fell within the target range, the target size remained unchanged. If performance exceeded 60%, the target was reduced to 60 × 60 cm and another five kicks were performed. If the success rate was below 40%, the target was increased to 80 × 80 cm, followed by five additional kicks. This adjustment process continued until performance stabilized within the desired range.

After the individualized target size was determined, participants completed 40 penalty kicks in the main session. These were divided into four blocks of 10 trials, with a 10-min rest period between blocks. Participants were instructed to focus on the target and aim for accuracy. They were allowed to use their usual kicking technique, and no specific attentional instructions were provided. The motor preparation phase was defined as the period immediately before movement initiation. EEG data were segmented into epochs time-locked to the onset of the kick, which was marked by an experimenter-operated trigger. Because only the pre-movement period was analyzed, EEG segments containing the overt kicking movement itself were excluded, thereby reducing contamination from movement-related artifacts (Li et al., 2025). Successful performance was defined as a trial in which the ball hit the target.

### 2.4 Subjective anxiety level

To control for potential changes in anxiety across the experiment (Li et al., 2025; Wilson et al., 2009), participants reported their subjective anxiety level during each rest period using a visual analogue scale ranging from 0 (“no anxiety at all”) to 10 (“highest anxiety level”). The anxiety ratings were analyzed across the four experimental blocks to determine whether anxiety changed during the task.

### 2.5 EEG recording

EEG was recorded from 64 electrode sites according to the international 10–10 system using an eego amplifier system (ANT Neuro, Germany) with a monopolar (referential) montage. Signals were referenced online to CPz and subsequently re-referenced offline to the average of the left and right mastoids (M1 and M2) for further analysis. AFz served as the ground electrode. EOG (electrooculogram) was recorded from electrodes placed above, below the left eye, the left, and right outer canthi. EEG signals were recorded with an online band-pass filter of 1–100 Hz and a 50-Hz notch filter. Data were sampled at 1000 Hz, and electrode impedances were maintained below 20 kΩ throughout the experiment.

### 2.6 Experimental procedure

Participants were asked not to consume alcohol- or caffeine-containing beverages for 24 h before testing. On the testing day, participants first received a brief introduction to the testing equipment and apparatus, provided written informed consent, completed basic demographic information forms, and performed the Trail Making Test Part A. They then completed a practice session while wearing the Lycra electrode cap to become familiar with the penalty-kicking task. During the practice session, the target size was adjusted individually until each participant achieved a kicking success rate between 40 and 60% (see 2.3.1). After the appropriate target size was determined, participants completed four experimental blocks of 10 penalty kicks, and before each block they were asked to report their anxiety level. A 10-min rest interval was provided between blocks. Throughout the experiment, participants were instructed to focus on the target and kick the ball as accurately as possible. They were asked to use their usual penalty-kicking technique and were allowed to take their normal number of preparatory steps before each kick. No additional attentional instructions were provided. The entire procedure lasted approximately 90 min to minimize potential fatigue-related effects on EEG recordings (Wang et al., 2021; Wang et al., 2019). The Football Penalty Task and the experimental procedure are illustrated in Figures 1a,b.

**Figure 1:**
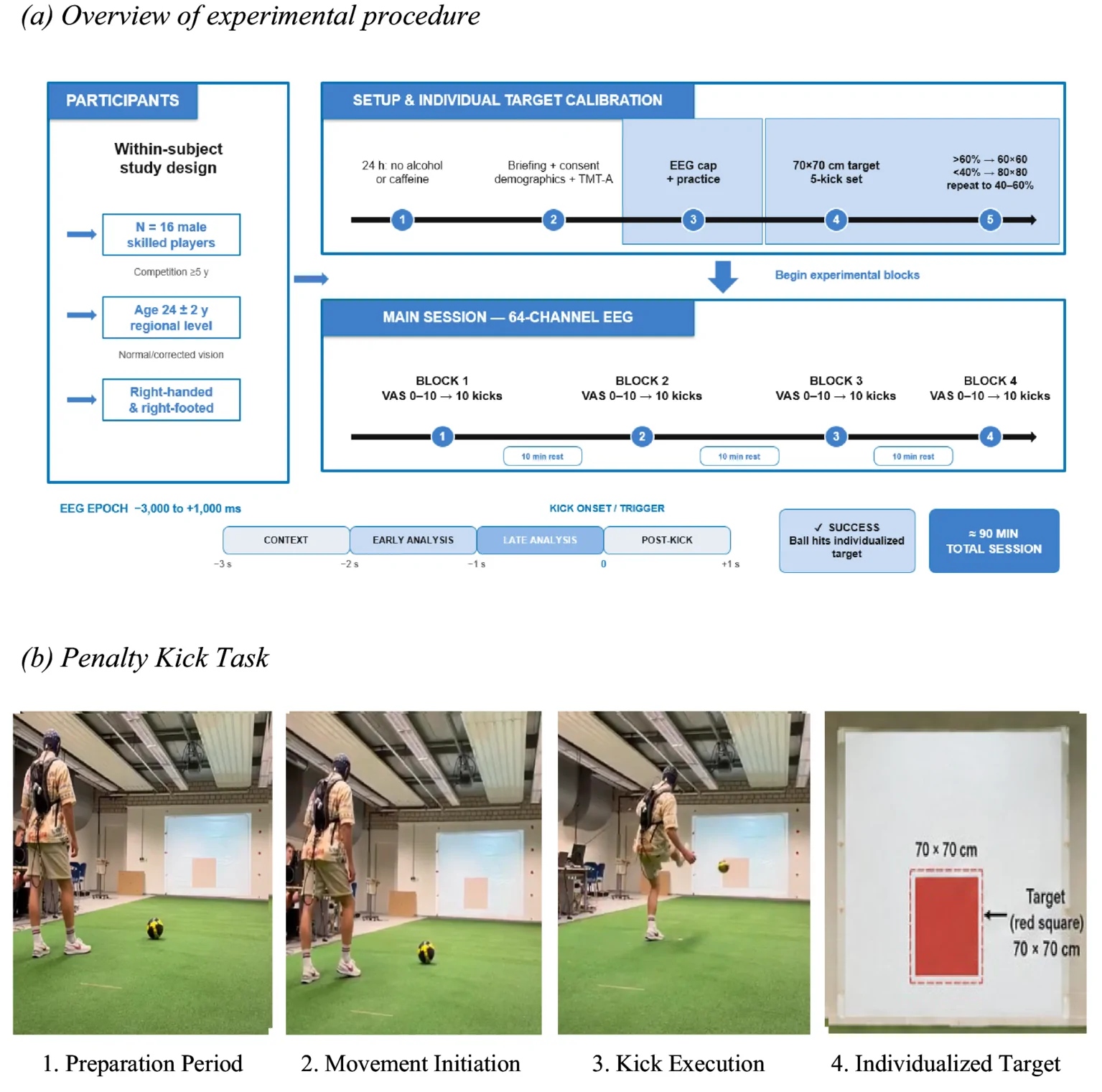
**(a)** Overview of experimental procedure. **(b)** Penalty Kick Task. **(a)** Schematic illustration of participant characteristics, experimental setup, individual target calibration, main EEG session, and the temporal structure of the analyzed EEG epochs relative to kick onset. **(b)** Representative images of the experimental setting during the football penalty task.

### 2.7 Data analysis

#### 2.7.1 EEG preprocessing

EEG data were preprocessed using EEGLAB functions (Delorme and Makeig, 2004) and custom MATLAB scripts. First, the data were re-referenced offline to the averaged mastoids (M1 and M2). Second, a finite impulse response band-pass filter from 1 to 30 Hz was applied. Third, EEG data were segmented into epochs from −3,000 to 1,000 ms relative to kick initiation. Channels with poor signal quality were identified and removed before artifact correction. The final 500 ms preceding kick onset were first visually inspected, and trials containing any residual artifacts were excluded from further analysis. Subsequently, trials containing amplitudes exceeding ±150 μV were rejected to minimize contamination from gross biological and movement-related artifacts. Independent component analysis (ICA) was then conducted using the Runica Infomax algorithm to identify and remove components related to eye blinks, eye movements, muscle, heartbeat, line noise, and channel noise recordings (Jacobsen et al., 2021; Wang et al., 2026). ICA has been widely used to separate physiological and movement-related artifacts from EEG signals (Delorme and Makeig, 2004) and remains one of the most commonly applied approaches for artifact attenuation in movement-related EEG recordings (Jacobsen et al., 2021). Removed channels were subsequently interpolated. To prevent differences in the number of trials from influencing coherence estimates, an equal number of artifact-free successful and unsuccessful trials was selected for each participant using random selection without replacement. The number of trials selected for each condition (successful vs. unsuccessful performance) was determined by the smaller number of available artifact-free trials for that participant (number of trials for each = 16). The cleaned EEG data were then segmented into two 1-s motor-preparation periods: an early period from −2,000 to −1,000 ms and a late period from −1,000 to 0 ms before kick initiation. For visualization purposes, electrode locations and statistically significant coherence connections were illustrated using a custom-built EEG Connectivity Editor, version 10.4, developed by Dr. KP Lab.

#### 2.7.2 Spectral coherence analysis

Fast Fourier transforms with a Hanning window were applied to all trials before coherence analysis to minimize spectral leakage. Coherence was defined as | *Cxy*(*f*) |^2^, where:


$$C_{\mathrm{xy}}(f)=\frac{\sum_{i}\{\mathrm{Xi}(f)-X(f)\}{\left\{\mathrm{Yi}(f)-Y(f)\right\}}^{*}}{\sqrt{\sum_{i}{|\mathrm{Xi}(f)-X(f)|}^{2}\sum_{i}{|\mathrm{Yi}(f)-Y(f)|}^{2}}}$$

and where *Xi*(*f*) and *Yi*(*f*) represent the Fourier transforms of the time series for electrode sites *X* and *Y*, respectively. Coherence was calculated in 1 Hz frequency bins and averaged across the appropriate frequencies to obtain the coherence values for the bandwidths. The electrode pairings of interest were the electrode pairings of T7–Fz, T7–Cz, T7–Pz, T7–Oz, T8–Fz, T8–Cz, T8–Pz, and T8–Oz in the frequency band 8–13 Hz. In addition, we applied a Fisher z-transformation to ensure an approximately normal distribution across subjects before conducting the statistical analysis.

### 2.8 Statistical analysis

Statistical analyses were performed using SPSS 26.0 (IBM). To test our hypotheses, a 2 (performance: successful/ unsuccessful performance) × 8 (coherence pairs: T7–Fz, T7–Cz, T7–Pz, T7–Oz, T8–Fz, T8–Cz, T8–Pz, T8–Oz) × 2 (time: −2,000 to −1,000 ms, −1,000 to 0 ms) three-way repeated-measures analysis of variance (ANOVA) was conducted. The assumption of sphericity was assessed using Mauchly’s test for effects involving coherence pair. When the assumption was violated, Greenhouse–Geisser corrections were applied to the degrees of freedom. *Post hoc* analyses were conducted following significant findings from the ANOVA. For all statistical tests, including *post hoc* comparisons, the alpha level was set at 0.05 prior to applying FDR corrections (Genovese et al., 2002). Effect sizes were estimated using the partial η^2^ statistic to quantify the proportion of variance explained by the factors in the ANOVA.

## 3 Results

### 3.1 Parameters of EEG coherence

The *Post hoc* comparisons are shown in Figure 2. Mauchly’s test indicated that the assumption of sphericity was violated for the performance × time × coherence site interaction, *W* = 0.015, χ^2^(27) = 52.104, *p* = 0.004. Therefore, Greenhouse–Geisser corrections were applied. The analysis revealed a significant performance × time × coherence site interaction for 8–13 Hz coherence, *F*(4.09, 61.41) = 3.328, *p* = 0.015, *η_p_^2^* = 0.182. Follow-up analyses revealed that the performance × time interaction at Fz–T7 was not significant, *F*(1, 15) = 0.672, *p* = 0.425, ηp^2^ = 0.043. However, a significant main effect of performance was observed at Fz–T7, *F*(1, 15) = 11.731, *p* = 0.004, *η_p_^2^* = 0.439. Pairwise comparison indicated that successful kicks were associated with significantly lower Fz–T7 coherence than unsuccessful kicks before action (*p* = 0.032, FDR corrected). However, significant performance × time interactions were also observed at Cz–T8, *F*(1, 15) = 15.773, *p* = 0.001, ηp^2^ = 0.513, and at Fz–T8, *F*(1, 15) = 9.108, *p* = 0.009, ηp^2^ = 0.378. *Post hoc* showed that only Cz–T8 successful kicks showed lower coherence during the −1,000 to 0 ms (*M* = 0.369 vs. 0.469 coherence, *p* = 0.04, FDR corrected).

**Figure 2:**
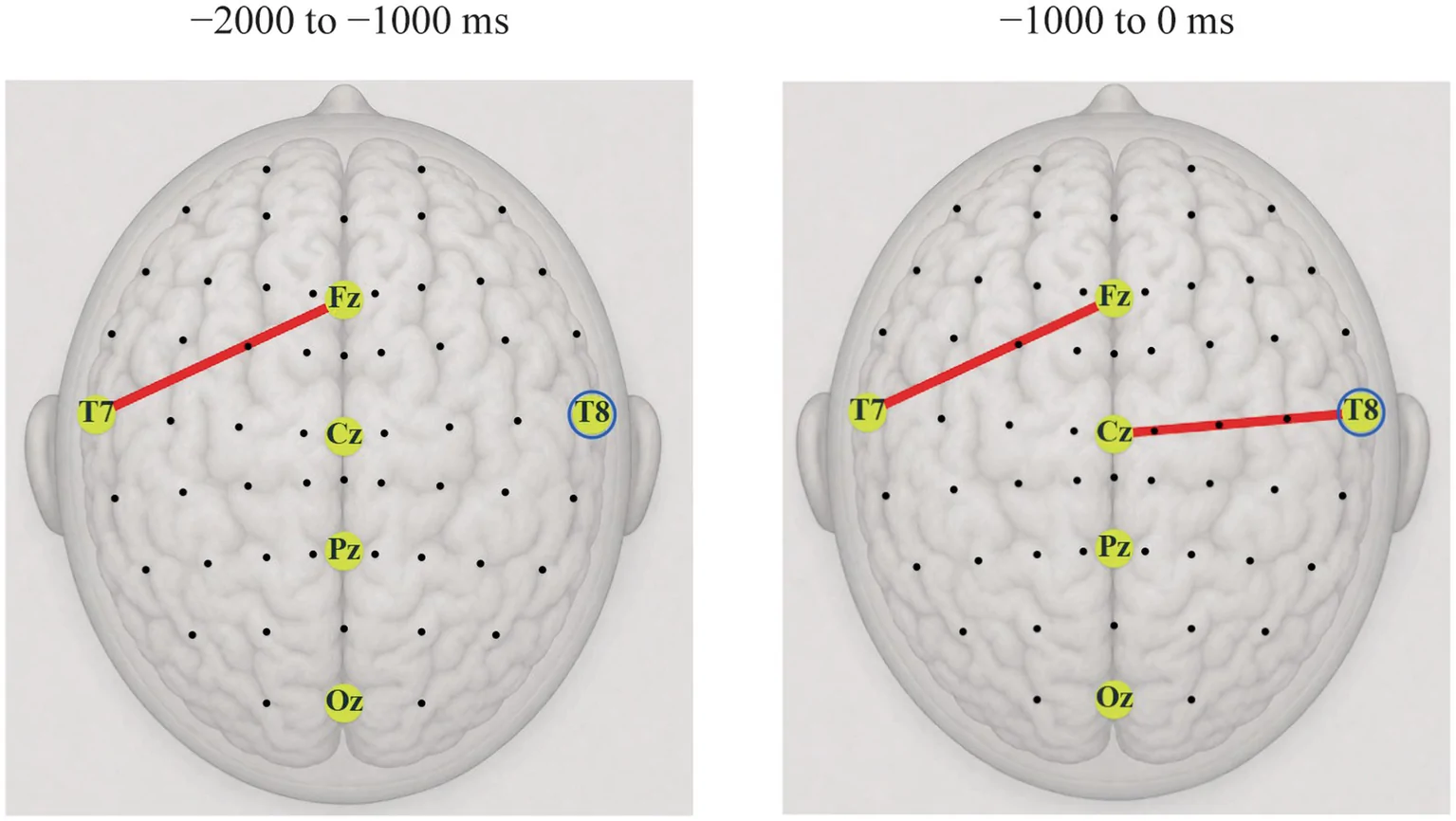
*Post hoc* comparisons. Nodes indicate electrode positions. Red lines indicate electrode pairs exhibiting significantly lower 8–13 Hz coherence during successful trials than during unsuccessful trials after false discovery rate correction. Highlighted labels identify the electrodes involved in these significant comparisons.

### 3.2 Control analyses

#### 3.2.1 Block-related changes in success rate

To assess whether a learning effect occurred during the task, success rates across the four blocks were compared using a one-way repeated-measures ANOVA. The analysis revealed no significant main effect of block, *F*(1.88, 28.20) = 2.047, *p* = 0.150, *η_p_^2^* = 0.120, indicating that success rates remained relatively stable across the four blocks. Therefore, no learning effect was observed.

#### 3.2.2 VAS anxiety level

VAS anxiety levels across the four blocks were compared using a one-way repeated-measures ANOVA. The analysis revealed no significant main effect of block, *F*(1.88, 28.24) = 1.306, *p* = 0.285, *η_p_^2^* = 0.080, indicating that anxiety levels remained relatively stable throughout the football penalty-kicking task.

## 4 Discussion

This study aimed to examine the temporal dynamics of 8–13 Hz alpha-band cortico-cortical coherence during motor preparation for successful and unsuccessful football penalty kicks performed under an individualized task-difficulty condition. The findings extend previous research on football penalty-kicking performance (Giustino et al., 2024; Li et al., 2025; Niźnikowski et al., 2025; Piskin et al., 2025; Slutter et al., 2021; Wilson et al., 2009).

and football-specific perceptual-motor and neurophysiological processes (Del Percio et al., 2019; Piskin et al., 2025). By using EEG coherence, the present study extends previous studies to the neural connectivity level by identifying time- and region-specific patterns of functional coupling associated with successful and unsuccessful kicking outcomes. The results support the psychomotor efficiency hypothesis and can also be interpreted within the multi-action plan (MAP) model. Successful kicks were characterized by lower Fz–T7 coherence across the entire −2,000 to 0 ms preparatory period and lower Cz–T8 coherence during the final −1,000 to 0 ms before kick initiation. Together, these findings suggest that successful football penalty kicking is associated with selective, time-dependent differences in functional coupling during motor preparation.

For Fz–T7 coherence, successful kicks consistently showed lower 8–13 Hz coherence than unsuccessful kicks across the entire −2,000 to 0 ms preparatory period. This result supports our hypothesis and aligns with previous studies indicating that lower 8–13 Hz coherence between the left temporal and frontal regions is associated with higher skill levels (Deeny et al., 2003, 2009) and better motor performance (Cheng et al., 2017; Wang et al., 2022a,b). The left temporal region is commonly linked to verbal–analytical processing, whereas the frontal midline region is involved in motor planning and attentional control (Deeny et al., 2003). Lower Fz–T7 coherence may therefore reflect reduced interaction between verbal–analytical processes and motor planning, suggesting less conscious monitoring or reinvestment in movement execution before successful kicks (Wang et al., 2022a,b).

This functional decoupling may allow skilled players to prepare the movement with less involvement of verbal–analytical and motor planning processes before the action. Importantly, the performance difference was present throughout the entire 2-s preparation period, indicating that successful performance depends on maintaining an efficient fronto-temporal network state rather than making a brief adjustment just before movement onset. These findings extend evidence of reduced cognitive–motor coupling from upper-limb precision tasks (Babiloni et al., 2011; Cheng et al., 2017; Deeny et al., 2003, 2009; Wang et al., 2022a) to a complex lower-limb action and identify reduced Fz–T7 coherence as a candidate neural correlate of efficient motor preparation in football kicking.

Contrary to our hypothesis, successful and unsuccessful kicks did not differ significantly in T7–Cz coherence during late motor preparation. Instead, successful kicks showed lower Cz–T8 coherence than unsuccessful kicks during the final −1,000 to 0 ms before kick initiation. The central region, including premotor and sensorimotor cortices, plays a key role in motor control (Cooke et al., 2014, 2015), while the right temporal region has been associated with visuospatial processing during motor preparation (Wang et al., 2021). Reduced Cz–T8 coherence may therefore indicate decreased coupling between motor control and visuospatial processes in the final stage of preparation (Deeny et al., 2003, 2009; Hatfield, 2018). One possible explanation is that successful performance during this late preparatory stage may not depend on reduced coupling between verbal–analytical processing and motor control. Instead, penalty kicking is a dynamic lower-limb action that requires the integration of target location, ball position, approach trajectory, postural control, and foot-to-ball contact within a precisely coordinated movement (Giustino et al., 2024; Piskin et al., 2025). Taken together, the reduction in Fz–T7 coherence and the later reduction in Cz–T8 coherence indicate that successful kicking is not simply associated with an overall decrease in cortical connectivity. Rather, it reflects a temporally structured and functionally selective reorganization of cortical communication, in which non-essential cognitive and motor processes are progressively disengaged as movement initiation approaches (Wang et al., 2022a). However, because the functional role of Cz–T8 coherence has been less extensively studied (Wang et al., 2022a) than that of Fz–T7 coherence (Cheng et al., 2017; Deeny et al., 2003, 2009; Gallicchio et al., 2017; Lo et al., 2019; Wang et al., 2021), this interpretation should be considered preliminary and warrants further investigation using direct measures of visuospatial processing and conscious movement control.

Overall, the findings of this study suggest that successful performance may involve a dynamic filtering process, thereby reducing noise within motor-related networks and facilitating efficient movement execution (Hatfield, 2018). The combined results of previous studies (Cheng et al., 2017; Deeny et al., 2003, 2009; Gallicchio et al., 2017; Lo et al., 2019; Wang et al., 2021; Wang et al., 2022a) and present studies enable us to delineate the “filter and fuel” characteristics (Wickens and McCarley, 2008) of attentional allocation, whereby the inhibition of irrelevant neural processes serves to decrease noise and optimize the allocation of neural resources to motor execution in lower-limb movements, in accordance with the psychomotor efficiency hypothesis (Hatfield, 2018).

In terms of practical implications for coaches and athletes, our findings may also be interpreted within the framework of the multi-action plan (MAP) model (Bertollo et al., 2016; Bortoli et al., 2012), which provides a useful approach for understanding how athletes achieve and maintain optimal performance. The MAP model defines four performance-related states: an optimal-automatic state (Type 1), an optimal-controlled state (Type 2), a suboptimal-controlled state (Type 3), and a suboptimal-automatic state (Type 4). According to this model, Type 2 and Type 3 states are typically accessed under conditions of stress, task difficulty, fatigue, or injury, whereas Type 1 and Type 4 states occur in less demanding situations. Given that the skilled football players in this study performed under a challenging task condition, our findings may correspond to the optimal-controlled (Type 2) state, in which performers maintain high levels of performance while exerting controlled attentional regulation. However, because performance trials were not explicitly classified according to MAP states, this interpretation remains tentative. Future research is encouraged to replicate the present study using a MAP-based classification approach or by experimentally manipulating stress levels to provide a more comprehensive understanding of optimal attentional allocation in sport performance.

This study has several potential limitations that should be considered. First, the sample consisted primarily of skilled football players, which may limit the generalizability of the findings to athletes of different skill levels or to novices (Chen et al., 2022a,b; Lu et al., 2025; Wang et al., 2020). Future studies should include participants with varying levels of expertise to determine whether similar neural patterns are observed across skill development stages. Second, the use of EEG coherence provides valuable information about functional connectivity but does not allow for precise localization of neural sources or causal interpretations of brain activity. Future research could incorporate multimodal neuroimaging techniques, such as fMRI or source localization methods, to better understand the underlying neural mechanisms (Slutter et al., 2021). In addition, using Neurofeedback to manipulate brain activity could provide causal interpretations of brain and motor performance(Chen et al., 2022a,b; Cheng et al., 2015; Cheng et al., 2024; Lo et al., 2025; Wang et al., 2023; Wang et al., 2022b; Yu et al., 2025). Third, the experimental design focused on a controlled penalty-kicking task, which may not fully capture the complexity and variability of real-game situations. Future studies should consider more ecologically valid settings, including dynamic and interactive environments, to examine how neural efficiency operates under realistic competitive conditions.

In conclusion, this study extends previous EEG research on football performance by showing that successful and unsuccessful penalty kicks are associated with distinct time- and region-specific patterns of 8–13 Hz functional coupling during motor preparation. Successful kicks were characterized by lower Fz–T7 coherence throughout the final 2 s before kick initiation and lower Cz–T8 coherence during the final 1 s. The results provide support for the psychomotor efficiency hypothesis by indicating that efficient performance is associated with selective reductions in cortico-cortical communication. Overall, the study highlights the potential importance of dynamic cortico-cortical reorganization in the preparation of successful football penalty kicks.

## Data Availability

The raw data supporting the conclusions of this article are available from the corresponding authors upon reasonable request.
